# Portal vein thrombosis in a patient on semaglutide

**DOI:** 10.5339/qmj.2024.75

**Published:** 2024-12-31

**Authors:** Mohammed F. Farooqi, Maria Khan, Arshad M. Muhammad, Adnan Agha

**Affiliations:** ^1^Internal Medicine, Tawam Hospital, Al Ain, UAE; ^2^Department of Internal Medicine, College of Medicine and Health Sciences, United Arab Emirates University, Al Ain, UAE; ^3^Diabetes & Endocrinology, Tawam Hospital, Al Ain, UAE *Correspondence: Adnan Agha. Email: adnanagha@uaeu.ac.ae

**Keywords:** Obesity, semaglutide, thrombophilia, type 2 diabetes mellitus, weight loss, venous thromboembolism

## Abstract

**Background:** Obesity and type 2 diabetes mellitus (T2DM) are modern-day pandemics that have a significant impact on global healthcare. The glucagon-like peptide-1 receptor agonist (GLP1-RA) semaglutide is a novel treatment for both tbl2DM and obesity, but can be associated with an increased risk of venous thromboembolism.

**Case presentation:** This case report describes a 59-year-old woman with tbl2DM who received semaglutide to manage glycemic levels, and also experienced the additional benefit of weight reduction. Within six months of initiating GLP1-RA, the patient experienced low back pain associated with nausea and poor oral intake. She had no known risk factors for venous thromboembolism or thrombophilia and had no history of significant illness in her family. Her physical examination revealed no significant findings. Only mild leukocytosis and neutrophilia were noted. She underwent an abdominal computed tomography scan, which revealed intrahepatic portal vein thrombosis without evidence of liver cirrhosis or abdominal malignancy. Her symptoms improved with oral anticoagulation (rivaroxaban). The result of thrombophilia examination was negative for inherited or acquired thrombophilia, except for a Janus kinase 2 mutation, which may increase the risk of thrombosis.

**Conclusions:** The use of GLP1-RA is increasing due to the growing desire for weight loss medications. Therefore, it is important that physicians better understand the possible risks of thrombosis before initiating GLP1-RA treatment.

## INTRODUCTION

Obesity is defined as the excessive accumulation of adipose tissue, either centrally or in the subcutaneous areas. It is classified using the body mass index (BMI), which is calculated as weight divided by height squared as follows: normal, 18.5–24.9 kg/m^2^; overweight, 25–29.9 kg/m^2^; and obese, ≥ 30 kg/m^2^.^1^ The World Health Organization declared obesity a healthcare crisis in 2017 as over two billion adults were overweight and 30% of them were obese.^2^ Glucagon-like peptide-1 (GLP1) is a hormone secreted by L cells in the ileum upon meal stimulation. GLP1 induces insulin secretion in relation to a meal with a low risk of hypoglycemia.^3^ Long-acting GLP1 receptor agonists (GLP1-RAs) are potent glucose-lowering agents and are effective in weight loss.^3^ The GLP1-RA semaglutide is 94% similar in structure to GLP1, with three substitutions to prolong its half-life. Semaglutide is administered as a weekly subcutaneous injection in patients with type 2 diabetes mellitus (T2DM) and/or obesity.^4^ Patients treated with semaglutide achieved glycemic control and weight loss in a recent clinical trial, with most patients achieving 5–10% weight loss.^5^ In the present case, a woman with tbl2DM and obesity was treated with semaglutide and developed portal vein thrombosis (PVT).

## CASE PRESENTATION

A 59-year-old woman with a history of tbl2DM presented to the emergency department with dull lower back pain that began after excessive household cleaning for 3 days. Apart from this pain, the patient complained of nausea and poor oral intake. The pain was not relieved by paracetamol. The patient denied any vomiting, diarrhea, changed bowel habits, or urinary symptoms. She previously had three healthy children with no history of venous thromboembolism (VTE), abortions, or thrombophilia. The patient had no significant family history other than parents with diabetes mellitus. The patient was not treated with hormone replacement therapy and never smoked or drank alcohol. Her medications included injectable semaglutide 1 mg once weekly (Thursdays), which she had started four months ago for diabetes control, and during this treatment she had lost 5 kg of weight.

Physical examination revealed the following: afebrile; heart rate, 80 beats per minute; blood pressure, 146/80 mmHg; oxygen saturation, 98% on room air; and respiratory rate, 17/min. She weighed 75 kg and had a BMI of 31.5 kg/m^2^. Her physical examination revealed no significant abnormalities, with no costal angle tenderness, calf tenderness, or abdominal findings. However, the patient continued to complain of low back pain, occasional moderate right quadrant pain, and non-resolving nausea. Her laboratory investigations revealed only mild leukocytosis and neutrophilia with normal liver and renal function tests (Table 1).

**Figure 1.**
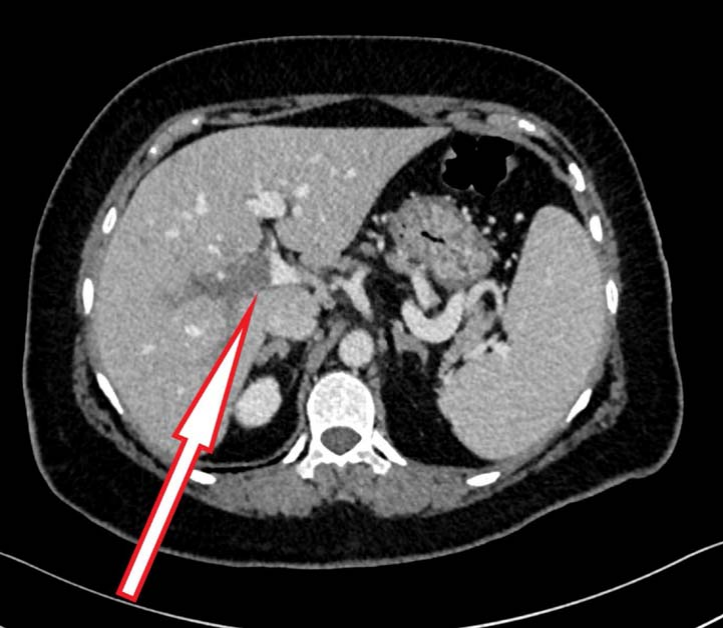
CT scan of the abdomen showing intrahepatic portal vein thrombosis (arrow).

**Figure 2.**
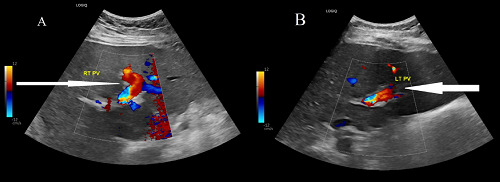
Abdominal ultrasound with liver Doppler of portal veins showing good blood flow in the right portal vein (indicated by a long white arrow) (A) and the left portal vein (indicated by a short white arrow) (B).

A contrast-enhanced computed tomography (CT) scan was performed to identify the source of the pain and rule out any obstructive gastrointestinal cause. The CT scan revealed evidence of intrahepatic PVT involving the right branch and part of the left branch, with no evidence of collection or mass (Figure 1). An upper esophagogastroduodenoscopy revealed mild gastritis. The patient was treated for gastritis with pantoprazole for two weeks, and oral anticoagulation therapy (rivaroxaban) was initiated. The patient showed clinical improvement within two weeks with increased appetite and reduced back pain and was discharged with a hematology follow-up to investigate the cause of thrombophilia. Hematological findings did not reveal any specific causes of inherited or acquired thrombophilia, although the patient had a Janus kinase 2 (JAK2) mutation that may increase the risk of thrombosis (Table 2). Abdominal ultrasound examination at the three-month follow-up showed recanalization of the thrombosed portal vein (Figure 2). The patient received anticoagulant treatment for six months, during which the symptoms completely resolved.

## Discussion

PVT is caused by thrombosis of the main portal vein or one of its branches, with or without extension into the mesenteric or splenic vein. It is categorized as malignant or benign based on etiology. Benign PVT usually develops in patients with portal hypertension secondary to liver cirrhosis and rarely occurs in patients with healthy livers.^6^ Benign PVT is rare, with an annual incidence of approximately four cases per 100,000 in the normal population.^7^ However, the incidence of benign PVT increases up to 11% in patients with liver cirrhosis.^8^ Benign PVT can be acute or chronic depending on the presence of collateral circulation and thrombus characteristics. Chronic PVT does not require treatment with anticoagulants.^9^ Up to 85% of patients with healthy liver status and benign PVT have an acquired or inherited prothrombotic condition.^10,11^ A recent meta-analysis of Asian individuals with benign PVT and healthy livers showed that thrombophilia was caused by protein C deficiency (10.7%), JAK2 mutations (8.5%), and antiphospholipid antibodies (7.5%).^12^ The JAK2 V617F mutation, a transversion mutation at nucleotide 1849 of JAK2, results in a valine-to-phenylalanine substitution at codon 617 and is associated with an increased risk of venous thrombosis due to endothelial overexpression of the von Willebrand factor and p-selectin. This mutation occurs in up to one-third of patients with benign PVT and healthy livers.^13,14^ A proportion of patients with JAK2 mutations develop myeloproliferative neoplasms (MPN), which are characterized by the proliferation of stem cells such as mature granulocytes (primary myelofibrosis), red blood cells (polycythemia vera), and/or platelets (essential thrombocythemia).^15^

Our patient had a JAK2 mutation with no evidence of MPN. This mutation may have contributed to the development of PVT. Obesity is a common risk factor for VTE,^16^ and although our patient had this risk factor, there was no evidence that this condition contributed to PVT. A recent meta-analysis also identified the use of GLP1-RA as a risk factor for VTE. Patients treated with semaglutide have a 266% (relative risk of 3.66) increased risk of developing VTE, raising concerns about the drug's suitability for use in individuals who are already at risk of VTE.^17^ Superior mesenteric thrombosis has also been reported in recent literature in a patient taking dulaglutide,^18^ another weekly GLP1-RA. However, to the best of the authors’ knowledge, there are no reports of PVT associated with GLP1-RA. Recent advances and increasing availability of long-acting GLP1-RA resulted in their widespread use, raising even more concerns about adverse events. In our patient, semaglutide initiation coincided with the development of PVT. Although the co-occurrence of PVT and initiation of semaglutide could be incidental, the patient had a JAK2 mutation without any overt MPN. A literature search revealed a similar case report. A 57-year-old male patient with tbl2DM taking the potent, long-acting GLP1-RA, dulaglutide, developed mesenteric vein thrombosis. This case highlights a rare but significant adverse event associated with GLP1-RA and the need to carefully monitor patients for unusual complications when initiating such therapy.

## CONCLUSIONS

Our case report describes a healthy middle-aged woman with no previous risk factors for thrombophilia, who developed PVT within six months of initiating GLP1-RA for weight loss and tbl2DM management. The patient was identified as having a JAK2 mutation without evidence of MPN. Based on this case report, healthcare professionals should be aware of the risks of VTE and PVT. An unusual clinical presentation of PVT may be associated with GLP1-RA. Therefore, an appropriate risk assessment should be performed before starting such treatment. It is important to remain vigilant in identifying and educating these patients about any signs and symptoms of VTE and/or PVT. This case report also reinforces the importance of ensuring that appropriate hematological information is obtained early and that thrombophilia screening, including testing for JAK2 mutation, is performed whenever a patient presents with benign PVT and a healthy liver. This case report is the first in the region to document an incidence of benign PVT in a patient with a JAK2 mutation treated with semaglutide.

### Competing interests

The authors have no conflicts of interest to declare.

### Informed consent and ethical approval

Informed consent was obtained from the patient and ethical approval was obtained from the Human Research Ethics Committee (MF2058-2024-1067).

## Figures and Tables

**Table 1:** Laboratory investigations on admission.

Laboratory test	Value	Normal range

Sodium	141 mmol/L	136–146 mmol/L

Potassium	3.6 mmol/L	3.6–5.1 mmol/L

Chloride	104 mmol/L	98–107 mmol/L

Bicarbonate	21 mmol/L	22–29 mmol/L

Creatinine	60 μmol/L	45–84 μmol/L

Urea	3.4 mmol/L	2.67–8.07 mmol/L

Albumin	36 g/L	>21 g/L

Calcium	2.33 mmol/L	2.23–2.58 mmol/L

Amylase	105 units/mL	28–100 units/mL

Lipase	45 IU/L	13–60 IU/L

Aspartate aminotransferase	24 IU/L	< 32 IU/L

Alanine aminotransferase	32 IU/L	< 33 IU/L

Hemoglobin	143 g/L	117–155 g/L

Mean corpuscular volume	81.7 fL	81–100 fL

C-reactive protein	37.9 mg/L	≤ 5 mg/L


**Table 2:** Laboratory investigations for thrombophilia.

Thrombophilia investigations	Results	Normal range

PT	12.1 s	9.5–12.5 s

INR	1.13	0.87–1.15

APTT	27.5 s	22.2–34.2 s

Fibrinogen	4.41 g/L	1.5–3.87 g/L

Factor V	71%	70–120%

Antithrombin III	101%	80–120%

Protein-C act	104%	70–130%

Protein-S free	76%	50–134%

D-Dimer	1.64 mg/L	0.129–0.523 mg/L

Cardiolipin IgG	< 2.6 CU	≤ 20 CU

Cardiolipin IgM	5.4 CU	

B2 Glycoprotein IgG	< 6.4 CU	≤ 20 CU

B2 Glycoprotein IgM	< 1.1 CU	≤ 20 CU

MDx Factor V Leiden	Not detected	

MDx Factor II (prothrombin)	Not detected	

MDx MTHFR	Heterozygous	

MDx JAK2 (V617F) mutation PCR	Detected	

Homocysteine total	9 μmol/L	12–15 μmol/L

Flow cytometry report	Negative for PNH	


PT: prothrombin time, INR: international normalized ratio, APTT: activated partial thromboplastin clotting time, Ig: immunoglobulin, B2: beta 2, MTHFR: methylenetetrahydrofolate reductase, JAK: Janus kinase, PCR: polymerase chain reaction, PNH: paroxysmal nocturnal hemoglobinuria.
